# Notes and Comments

**Published:** 1922-05

**Authors:** 


THE
### HOSPITAL and HEALTH
REVIEW

### THE BRITISH MEDICAL ASSOCIATION AND THE VOLUNTARY HOSPITALS.

FOR some years the organisation of the voluntary hospitals, particularly as affected by patients for whom payment is made, by a contributory scheme or otherwise, has been considered by the British Medical Association and resolutions have been adopted on the subject. The Council of the Association has recently made a report for submission to the Representative Meeting which will take place in Glasgow this summer. The report aims at the consolidation of the known policy of the Association and many of the details have been adopted at previous Representative Meetings.

It begins by pointing out that the voluntary hospitals have grown up upon a general understanding between the subscribers, the management and the honorary medical staff, that both funds and services are given gratuitously to patients unable to pay for adequate medical treatment. The present general tendency to exact payment, however small, necessarily modifies the original understanding and " fundamentally alters the basis of the relationship between the honorary medical staffs and the subscribers."

The Association recognises a dual policy as regards the voluntary hospitals : (*a*) that the purely charitable side should be continued wherein indigent patients are maintained by gratuitous contributions and treated gratuitously by the honorary medical staffs ; (*b*) that patients who are not indigent may be received for treatment at voluntary hospitals when adequate treatment cannot be obtained elsewhere, and that for them payment should be received by the hospital, either from the patients themselves or on their behalf from the authority or body referring them to the hospital, and that on account of their treatment some method of remuneration of the honorary medical staff should be arranged.

In view of the tendency of the State through local and central authorities to require services of the voluntary hospitals, the Association holds that in any arrangements for such services the State should pay the full cost to the hospital, so that no portion shall be charged to the charitable funds of the hospital, and that the payment so made shall include an amount for the remuneration of the honorary medical staff. The Association considers it neither just nor expedient for the community to throw upon one section the onus of undertaking, gratuitously, professional work. Services rendered to the State should be remunerated by the State. Such work could be requested gratuitously only as an act of charity or as a demand on the services of the citizen. Charity can be requested only on behalf of those who are themselves unable to pay, and the State does not come within this category.

On the other hand, the Association admits that the voluntary method of administration is to the advantage of the public, medical science, and the medical profession, and should be maintained. It holds that the essence of the voluntary hospital system is the independent and voluntary management, and that this is not necessarily related to the conditions of service of the medical staff. While agreeing that contributions may be either gratuitous or may be made for services rendered or to be rendered, the Association considers it undesirable that the voluntary hospitals should be subsidised by the local rating authorities, except in so far as payment is made for patients for whom they are responsible. Gratuitous contributions by employers or employees should not be considered as the payment of premiums for sickness or accident, nor as entitling the contributors to claim hospital treatment either for themselves or for persons nominated by them, but as charitable contributions to be expended at the discretion of the managers of the hospital. Every patient who is able should make a contribution towards the cost of maintenance and treatment, unless the contributory method of subscription is adopted as essential in industrial areas.

In any schemes for providing benefit by voluntary hospitals, the Association thinks it undesirable that the hospitals should undertake any insurance risk—*i.e.*, undertake to provide hospital benefit, when required, in return for a periodic payment by an individual or a group of individuals. When schemes are set up to provide payment for hospital benefit for specified individuals or groups of individuals, such schemes should be organised not by the hospital, but by some independent body. The acceptance by voluntary hospitals of an insurance risk would (*a*) prejudice the primary consideration in the admission of a patient to hospital, namely, suitability for admission on medical grounds; and (*b*) in the event of the actuarial estimates upon which the scale of premiums and benefits are based proving faulty, render the hospitals liable to meet outlays for which there was no financial provision. The primary consideration in the admission of a patient to hospital is held by the Association to be suitability on medical grounds. Some means of investigation into his circumstances by means of an almoner or other agent should be employed.

Hospital patients should be classed under the following groups: "Free" (Indigent), "Tariff," and "Private." The first comprises those certified by the almoner or other officer of the hospital as unable to contribute in any way towards their maintenance and treatment; the second, those paying, or for whom is paid, in part or in whole, the tariff cost of maintenance and treatment; this group includes all those paid for by public authorities approved societies, employers, insurance companies, or other bodies, or under any contributory scheme; and the third, those who pay for special accommodation and arrange for medical treatment fees independently of the hospital. Cases of obvious destitution or cases already in receipt of poor-law relief, after they have been seen once in the casualty or out-patient department, should be referred to the poor-law relieving officer, unless retained by the hospital for treatment. But in case of patients referred by poor-law medical officers to hospital for consultation or treatment, payment should be required from the poor-law guardians for both maintenance and treatment.

When a voluntary hospital enters into a financial arrangement with an employer, public authority, approved society or any other body for the reception of patients, the Association holds that such arrangements should be taken to cover the full cost of maintenance and treatment according to an agreed tariff. Inability to pay for adequate treatment as a private patient whether in the hospital or outside should be the consideration for the admission of all tariff patients for hospital treatment. All persons, whether insured or not, whose income from all sources does not exceed the limits of a specified scale should be eligible for hospital benefit on tariff rates. The following scale is suggested, subject to economic and local variations:—

CLASS 1.—*Limit of Income* £250.
- (a) Single persons over 16 years of age.
- (b) Widow or widower without children under 16 years of age.

CLASS 2.—*Limit of Income* £300.
- (a) Married couples without children under 16 years of age.
- (b) Persons with one dependent under 16 years of age.

CLASS 3.—*Limit of Income* £350.
- (a) Married couples with a child or children under 16 years of age.
- (b) Persons with more than one dependent under 16 years of age.

Persons whose income places them beyond the specified scale arrangements may be received as private patients. The Association holds that no tariff patient should be admitted without the recommendation of a private practitioner, except in emergency. Of the payment made by or for tariff patients it asks for a percentage to be passed into a fund at the disposal of the honorary medical staff.

We have given a very full summary of this Report, because it embodies the views of the one body which can claim to represent the medical profession. Moreover, they have been endorsed by a conference of representatives of the voluntary hospitals, summoned by the British Medical Association. On the whole they are reasonable, but some professional bias is to be expected. The definition of a voluntary hospital as one under voluntary management is surprising. Support by voluntary subscription has always been regarded as the essential point, and the legend on many voluntary hospitals bears this out. Voluntary management is a mere corollary to this. The definition of the Association is like an attempt to make a pyramid stand on its apex instead of on its base. The idea appears to be to alter a well-recognised meaning in order to promote a policy—an arbitrary procedure.

The demand that the medical staff should receive part of the contributions made by or on behalf of patients is reasonable, subject to limitations. The Association's description of the work of the medical staff as "gratuitous" overlooks the fact that hospital appointments are valuable things which are eagerly competed for by men of the highest qualifications. They are the one channel to the rank of consultant, with fees a considerable multiple of those of the ordinary practitioner. The hospital work of the consultant is a case of the rich paying for the poor, and the system has worked well, and to the benefit of all concerned. It has been pointed out by Lord Cave's Committee, and by members of the medical staffs of the large hospitals, that the carrying of a percentage of payments for the maintenance of patients to a staff fund would check subscriptions and endanger the whole voluntary system. But when the payment exceeds the cost of maintenance the claim to a percentage is justified. This line of demarcation was supported by a considerable minority at the conference of the representatives of the medical staffs of the hospitals. We think it unfortunate that it was not accepted. Patients who can only contribute towards maintenance in a hospital or barely pay for maintenance are fit subjects for charity. On the other hand, those who can do more than this belong to the class on which the operating surgeon and other specialists rely for a livelihood. A large part of this class has become the "new poor." Crushed by grinding taxation on the one hand, and the increased cost of living on the other, they cannot pay the increased charges of the nursing home and for treatment which, as time goes on, tends to be more elaborate, and therefore more expensive. But the operating surgeon, too, has had living made much more difficult for him, and it is only fair that when patients of this class are driven to the hospital, where he has to treat them, he should be recompensed according to their capacity.

In laying down the limits of income in the scale of charges the Association has endeavoured to judge a patient's capacity to pay by two factors—income and liability for dependents. But the attempt is rather crude. A man with six children is treated exactly the same as a man with two, although the former's capacity to pay is obviously much less than that of the latter. Children are the only dependents taken into account. The Association appears to have forgotten aged dependents, although they are very common, and often impose a more prolonged burden. We would suggest that a lesson in this matter might be taken from the income tax authorities.

What we consider the most objectionable of all the proposals of the Association is that, excepting in emergency, a tariff patient should not be admitted to hospital without the recommendation of a private practitioner. Co-operation between the practitioner and the hospital to which he sends his cases is most desirable, both in the interest of himself and of his patients, but that persons outside a hospital should have the power to forbid access to patients would be nothing short of tyranny. Hospitals exist largely because of the superior skill and equipment which they provide for difficult cases, and these come into play in deciding suitability for admission. No one should be allowed to bar the patient's resort to them. Unfortunately, it is a constant experience that cases are sent in too late. The veto demanded would tend to increase this evil.

### SIR PATRICK MANSON.

THE death of Sir Patrick Manson has removed a great benefactor to mankind, on whose work tropical medicine is based. Great men there were before him who described tropical diseases and, in some cases, the parasites which caused them. But of the conveyance of these diseases by insects nothing was known, though it is true that surmise was not wanting. Consequently, prevention was entirely lacking. Of the adage "Truth is stranger than fiction," no better example can be given than Manson's work. No romance of more enthralling interest than the discovery of the origin of malaria was ever penned. When Manson began practice in the Far East in 1866 tropical medicine was in its infancy, and he felt acutely his ignorance of the diseases with which he was confronted. He at once set to work to fill the gap. One disease very prevalent was elephantiasis. Another pioneer in tropical medicine, Timothy Lewis, had discovered that a minute worm, which he called *Filaria sanguinis hominis*, often existed in the blood of the natives of certain districts of India, and was associated with elephantiasis. In China Manson found the parasite in the blood of a large proportion of the inhabitants of certain parts. As it showed no evidence of growth while in the blood or any features indicating that it was capable of reproduction, he inferred that it was an immature form. This he found to be true, but he had been anticipated by Bancroft as well as by Lewis. However, the problem remained, How should the parasite gain access to the human body ? He argued that, as it was never found in any of the excreta, it could not escape spontaneously. There must be some carrying agent capable of penetrating the skin and absorbing blood. It struck him that the most likely one was the mosquito. He then found that the filaria was often present in blood taken at night and seldom present in that taken by day. This periodicity at once suggested adaptation to the habits of the mosquito. He then set to work to find whether the filaria was ingested with the blood which the mosquito sucked. He found that it was, and that it flourished in the digestive juices of the insect. He traced it through the stomach wall into the abdominal cavity and thence to the thoracic muscles. During this process the filaria grew and developed into a mature form capable of reproduction. Manson conjectured that when the mosquito died the filaria escaped into water and thus reached the human body. This discovery of two stages in the life of the parasite—one passed in man, the other in an insect—was of far-reaching importance and revolutionised the pathology of tropical diseases both of man and animals, for it proved applicable to the most important ones. Manson's next stroke of genius was the extension of his discovery to malaria. It is true that an insect-borne origin had been previously suggested, but it was only a surmise and had even been forgotten when a French investigator, Laveran, in 1880 discovered in the red blood corpuscles the parasite of malaria. Manson pointed out the close parallelism between the conditions of the evolution of this parasite and of the filaria. He reasoned that as the latter was enclosed in a red corpuscle and incapable of leaving the body spontaneously, some blood-sucking animal must be the agent of transmission. He had not the opportunity of testing this hypothesis, but he inspired Sir Ronald Ross to do so and suggested that, failing human subjects, he should work out the life-history of the corresponding parasite in birds. This Ross did, and his work culminated in the establishment of the mosquito-borne origin of malaria in man. On that doctrine depend the measures which have proved so successful in eradicating malaria. Manson was thus not only the father of modern tropical medicine, but of tropical hygiene.

---

### THE HOSPITALS COMBINED APPEAL.

PUBLIC lists of subscriptions for the great combined appeal on behalf of the hospitals of London were opened on April 18 at about 950 banks in the metropolitan area. Local committees and " lists-in-aid " are now being arranged.

The Lord Mayor of London, Sir J. J. Baddeley, will open " The Lord Mayor's City of London List-in-Aid " on May 1, and will preside at a Guildhall dinner on behalf of the Combined Appeal, provisionally fixed for June 26 or 27.

The objects of the appeal are to raise new money, and contemporaneously to build up new sources of permanent income by means of regular contributions from wage-earners on lines that have proved so successful in the provinces. Regular subscribers and donors to individual hospitals are requested not to postpone or withhold their customary or intended direct support. No advantage will result if the combined appeal is to displace ordinary revenue. It is sought to ensure that the results of this effort shall be additional, and without prejudice to usual or standard benefactions.

The plan of campaign provides that each hospital shall be at liberty to continue its separate propaganda until May 24, after which date, for a minimum period of two months, all will act in accord with, and as part of, the central organisation. Correspondence concerning renewal or acknowledgment of donations due or received direct by any hospital during the period will proceed without interruption, but no appeal for new money will be made except from the central organisation. No local flag days are to be sanctioned by the organising committee between May 25 and July 24.

New money raised will be applied without delay (1) to the reopening of closed beds ; (2) to help to meet the higher costs of materials, drugs, instruments, &c., until prices become more normal ; (3) to provide re-equipment which is now lacking, but essentially needed ; and (4) to reduce floating debt. A prompt response will also enable claims to be made good for the Government grant which is conditional upon the raising of new money on a " pound for pound " basis. All communications in respect of the organisation should be addressed to the Director-General, Combined Hospitals Appeal, 19 Berkeley Street, W.1.

### THE EUROPEAN EPIDEMIC MENACE.

THE resolution passed recently by the League of Nations Sanitary Conference at Warsaw contains a warning which has been unheeded by the majority of the public in Western Europe. Experts gathered there came to the conclusion that the increasing prevalence and extension of cholera in the Ukraine combined with the mass migration towards the north-west from that and from other famine-stricken areas, constitute an immediate danger to the rest of Europe. " These adverse conditions are increasing rapidly, and the situation is becoming more menacing." The Conference was convinced that much greater efforts were necessary if far more serious suffering and death were not to ensue amongst the population of the infected areas. It considered that the difficulties accentuated by the lack of medical men and trained personnel were likely to impede reconstruction and hamper trade.

This Conference was attended by representatives of the European members of the League of Nations and by Germany, Soviet Russia, Soviet Ukraine, Hungary and Turkey. As is well known, the League of Nations has been co-operating on a large scale with the health authorities of Poland during last year through the League's Epidemics Commission, the head of which is a distinguished public health officer with long Indian experience, Dr. Norman White. Various Governments have contributed over £130,000 for the anti-epidemic campaign, and the League of Red Cross Societies £10,000, besides 280,000 suits of underwear and 38,000 pairs of rubber gloves. But these resources have proved to be quite inadequate to combat the armies of diseased persons spreading infection. The incidence of typhus alone has risen in Russia from some 90,000 cases per annum before the war to considerably over 3,000,000 in 1920.

The Russian revolution, with the breakdown of administrative machinery that accompanied it and the successive wars and civil wars and migrations, has been followed by the scattering of typhus and relapsing fever through the whole of Eastern Europe. Thanks to the work of the Epidemics Commission, by the summer of last year the situation appeared to be well in hand ; the amount of disease was diminishing and the staff was well able to handle the stream of refugees and re-emigrants. But the Russian famine of last autumn drove hundreds of thousands of peasants westwards seeking for food.

It is expected that by next August the famine will have reached its climax, and as the warm weather returns the number of emigrants increases. Cholera has already reappeared and will spread. This is the situation that governs European reconstruction to-day, for unless Russia is healthy, business on any large scale becomes impossible. In fact, the foundation of the Russian economic revival, discussed in detail at Genoa, is a public health problem. The peoples of the West ought to realise the epidemic menace of the East, for sacrifices may be called for in the near future in order to stem the onward rush of disease.

### THE REMOTE EFFECTS OF DENTAL DISEASE.

THE teaching that a small focus of infection in the body may be responsible for an almost infinite number of diseases, including rheumatism and Bright's disease, undoubtedly contains a considerable element of truth. But like all teachings, it is liable to parody. Recently a publication by Prof. Schottmüller, of Hamburg, has called a halt to the wholesale extraction of teeth as a remedy for gastric ulcer, cholecystitis, appendicitis, climacteric disturbances, hypochrondria, arteriosclerosis, and a host of other diseases. There may, of course, be some connection between dental abscesses or pyorrhœa with these diseases, but if making a clean sweep of the teeth becomes a gene al practice in the hope that a bow drawn at a venture may secure a hit, a reaction must follow to the detriment of a teaching which may be sound in principle. According to Prof. Schottmüller it is as much as their (natural) teeth are worth for travellers in the U.S.A. to consult doctors about any of the diseases which have recently been traced to infectious conditions of the mouth. He paints a lurid picture of neurasthenic patients falling into the hands of fanatic disciples of the doctrine of focal infection, who, with a mania for filching teeth, tonsils, appendices and other appurtenances, also filch what little vitality these patients once possessed. At his hospital in Hamburg more than 10,000 bacteriological examinations of the blood have been undertaken in cases suspected of septic infection from some focus in the body, but not in a single case have germs cultivated from the blood been tracked to a dental abscess. Anyone familiar with the difficulties of tracking germs in septicæmia to their source will note with surprise that Prof. Schottmüller claims invariably to have found the septic focus or site of entry of germs when blood cultures have been positive. He has undoubtedly done useful work in ridiculing extremists, but he seems to have overshot the mark. Many patients have at least been partially restored to health by the kind offices of the extracting dentist.

---

### THE NEW GERMAN VENEREAL DISEASE LAW.

FOR some years the German Anti-Venereal Disease Society (D.G.B.G.) has been agitating for a new and comprehensive anti-venereal disease law which should give expression to the considerably changed opinions of the authorities. The draft of the new law has been under discussion for some time, and its passage through the Reichsrat has not proved uneventful. It has, however, survived its critics, and it promises to become an effective weapon against persons who have hitherto been able to disseminate venereal disease with comparative impunity. The new law provides for as much as three years' imprisonment for the offender who propagates venereal disease, knowing or suspecting that he or she is in an infectious state. Another important clause deals with continuity of treatment. This is to be free to all who cannot afford to pay, but the benefit of free treatment carries with it the obligation to submit to it as long as the medical authorities consider necessary. The corresponding English Society, the N.C.C.V.D., is agitating for the provision of continuous treatment. Its hands will be strengthened by the fact that this has been made compulsory in Germany. It would be difficult to overrate the importance of this measure, as the failure of specific treatment in the past has been largely due to patients absenting themselves from treatment when they, but not their medical attendants, were satisfied that complete recovery had been effected. The German law also provides penalties for the exposure of persons to infection ; if a child, suffering from venereal disease, is put out to nurse by persons who conceal this fact, or if its guardians allow a syphilitic infant to be suckled by a healthy wet-nurse, concealing the nature of the infant's disease, penalties may be inflicted. Provision is also made for wet-nurses to hold health certificates, and for their employers to insist on the examination of such certificates. Much harm has been done in Germany by unqualified practitioners who profess to cure venereal disease. The new law provides penalties both for the quack and for the medical practitioner who undertakes treatment by correspondence. Other clauses provide for the control of offensive and misleading advertisements. A striking feature of the new law are the obligations imposed on the doctor who undertakes to cure venereal disease. He must give each patient full instructions about the nature of the disease and how to prevent its further spread. In the case of patients under age such instruction must be given to the patient's parents or guardians. The doctor must also give the patient a résumé of the new law and explain its penalties. Thus, though the competition with the quack promises to be eliminated, the duties and responsibilities of the medical practitioner are considerably increased.

---

### LUNACY REFORM.

THE inquiry into the treatment of mental disease is making steady progress at the Ministry of Health, and Sir Cyril Cobb, the Chairman of the Committee, is to be congratulated on the unprejudiced way in which he is obtaining evidence from a variety of sources. It is becoming clear, however, that this inquiry is so limited that in the near future an appropriate body will have to examine the whole question of the Lunacy Laws. In fact, this was recently admitted by Sir Alfred Mond. In the meantime, much of the jungle which smothers this subject will have been cleared away by the work of three useful Committees that have been appointed by the Board of Control with the approval of the Ministry of Health.

The first of these is to consider the question of diet in county and borough mental hospitals and is to report on the changes, if any, which are desirable, and whether a minimum dietary scale should be fixed. Sir R. W. Branthwaite, C.B., a Commissioner, is the Chairman. A second Committee is to consider the clinical and other records and to report in what ways the system of keeping these records can be improved. Mr. A. Rotherham, M.B., is the chairman. The third Committee has been appointed to consider the nursing services in county and borough mental hospitals, with Dr. C. Hubert Bond as chairman.

There is an agitation in certain influential quarters for a Royal Commission to be appointed to inquire into the many charges that have recently been made against our system of dealing with mental illness. Such a Commission is necessarily a cumbersome method, taking time, involving public expense and too fond of producing Blue Books which collect dust on our shelves without effecting any real result. The appointment of small Committees composed of experienced men, with specific and detailed terms of reference, is a much more practical way of improving the treatment of those who suffer from mental disease than a dozen Royal Commissions.

Sir Alfred Mond recently stated that he was considering legislation on lunacy matters. Although no further official information is available, it is hoped by the advance guard of lunacy reformers that facilities will be afforded for the treatment of independent cases of mental trouble without certification. The Lunacy (Scotland) Act of 1866 has enabled such cases to be treated and frequently cured. If the Act has worked satisfactorily north of the Tweed for nearly sixty years, there would appear to be no reason why similar opportunities should not be given to residents in England and Wales to be treated in the earliest stages of mental diseases away from the licensed house and without a certificate. If Sir Alfred Mond proceeds on such lines, he will be acting wisely, and will help to reduce the wastage of lunacy.

---

### THE NATIONAL COUNCIL FOR MENTAL HYGIENE.

A provisional committee consisting of leaders of the medical profession, and mainly of those interested in medical psychology, has decided to form a National Council for Mental Hygiene. The recent establishment of a few hospitals and clinics for the treatment of functional nervous disorders affords evidence of the fact that the early recognition and treatment of these maladies is now regarded as important. There are, moreover, many societies and associations, old and young, engaged in promoting the study of mental disorders, the welfare of the insane, the problems of industrial psychology, and the various aspects of mental deficiency. The object of the Council is to encourage these institutions and societies to expand and to add to their usefulness by organised co-operation. The Council will also help to establish psychological clinics at general hospitals for the treatment of early mental and nervous disorders. It will endeavour to make mental hygiene a more prominent subject in the education of medical students, and by instructing the public in the principles underlying mental health and illness diminish the enormous waste of time and energy in all classes of society which now results from widespread ignorance concerning these questions. A similar organisation, under the name of the National Committee for Mental Hygiene, has been established for some years in the United States.

### AUTO-SUGGESTION.

THE enormous publicity devoted to M. Coué's lectures by the daily Press, always eager for the sensational, has given the public an exaggerated idea of their importance. He has nothing new to offer us, for the value of suggestion in the treatment of functional nervous diseases is now widely recognised. But he is transparently honest, and has a most engaging and impressive personality, and hence his success. To persuade neurotic or hysterical persons that they are better, or to induce them to persuade themselves that they are better, is to go a long way towards cure. M. Coué's method is to get them to repeat in a monotonous voice : " Day by day, in every way, I am getting better and better." But successful though this method has proved in his hands, it is only one form of psycho-therapy, and touches only the fringe of a subject which has in recent years received great attention in this and other countries, and is undergoing great development, as is shown by the review which we publish elsewhere (p. 227), as well as by the formation of the National Council for Mental Hygiene described in the previous article.

---

### POISONING THROUGH THE SKIN BY MARKING INK.

SEVERAL small outbreaks of poisoning by marking ink have lately been reported in foundling homes in Germany where the marking of babies' napkins with ink, not rendered harmless by a preliminary washing of the newly-marked napkin, has led to alarming symptoms. Probably many such cases are overlooked ; little was known of this danger till about a year ago, and now at least three papers have appeared in the German medical press showing that the risk of serious poisoning by marking ink is far from negligible. There are two main groups of marking inks, those which contain metals, such as silver, which turn black on reduction, and those which contain organic colouring matter, such as anilin black. Before the war the first group was favoured almost to the exclusion of the second, but the rise in the cost of silver and other metals has given an impetus to the manufacture of the organic marking inks. These are probably quite harmless, provided the directions given with each bottle are carefully followed and the newly-marked linen is not worn before it has been washed at least once. It is not yet quite clear what the substance is which has given rise to symptoms of severe poisoning. Some authorities trace them to nitro-benzol poisoning, but in the latest publication on the subject, anilin black, in the form of the anilin-chlorhydrate, has been held responsible. These differences of opinion are, however, chiefly of academic interest, as the action of nitro-benzol and of anilin black on the body is practically the same. Both are blood poisons which produce methæmoglobin, the presence of which is betrayed by the dusky colour of the patient's lips and skin. Other symptoms of this form of poisoning in infants are sudden collapse and marked weakness of the pulse.

### THE INTERNATIONAL COMMITTEE ON DISABLEMENT.

IMPORTANT decisions on questions of interest to men maimed either on the battlefield or in the workshop were taken by the International Committee of experts on disablement which recently met at Geneva. The meeting was attended by representatives of organisations of ex-service men and official bodies or departments concerned with disablement in France, Great Britain, Italy, Poland, Germany and Austria, and by representatives of the Health Section of the League of Nations and the League of Red Cross Societies. The British Legion was represented by Major J. B. Cohen, M.P.

The Committee considered it urgently necessary that the experience in prosthesis and orthopædy gained in various countries should be made more widely known, in the interests of those who are disabled in industry or in war. This task would best be achieved by a single institution of an international character, which could collect information, present it scientifically, and, as an immediate practical step, organise an international exhibition of artificial limbs and other orthopædic instruments. It should work in close and continuous collaboration with existing national administrative departments, disabled men's associations, medical organisations and other bodies directly interested in such problems. Having regard to the fact that artificial limbs and orthopædic instruments are generally intended to increase the capacity for work of disabled men, this undertaking could properly be entrusted to the International Labour Office, which was therefore invited to take up the task. Legislation concerning industrial accidents should be amended in such a way as to allow disabled men the benefit of artificial limbs and orthopædic and medical treatment for an unlimited period. Meanwhile, liaison should be established between departments dealing with the supply of artificial limbs and orthopædic instruments to disabled ex-service men on the one hand and the social insurance organisations on the other, and these departments should to some extent be maintained permanently to deal with the needs of persons disabled in industry. Disabled ex-service men living in countries other than their own should be provided with artificial limbs and orthopædic and medical treatment. Pending the conclusion of an international convention, this subject should be dealt with by reciprocity agreements between States on clearly defined lines. The International Labour Office was requested to communicate these conclusions to all Governments.

With regard to the proposal for an international exhibition showing the progress made in the manufacture and use of artificial limbs, as an exhibition in one town, such as Geneva, would be inaccessible to the majority of those interested, the Committee favoured the suggestion of a travelling exhibition. It was suggested that when the material had been got together by the League of Red Cross Societies with the assistance of the International Labour Office and the Health Section of the League, the exhibition should be lent to each of the national associations of disabled men in turn.

### THE SMOKE EVIL.

THE Minister of Health has already arranged for the appointment of one or more competent advisers with a view to assisting local authorities and manufacturers in combating the smoke evil. It will be remembered that this was one of the recommendations made by Lord Newton's Committee. This Committee was the first official inquiry held into the subject of smoke abatement for nearly seventy years, and its report showed clearly that a great deal of the smoke from factories and commercial premises is unnecessary and should be prevented.

The general law in regard to the abatement of smoke nuisances dates back to the Public Health Act of 1875. The inadequacy of this Act is proved by the facts that such municipalities as Manchester, Sheffield, Leeds, Liverpool, Nottingham and Glasgow have been forced to apply to Parliament for special powers to deal with the nuisance. All students of public health realise that smoke is an unhealthy and unnecessary evil. It leads to immense waste of fuel, and the destruction of buildings and property of every kind which it involves represents many millions a year. In residential London, domestic smoke is responsible for two-thirds of the problem.

This is an evil that it is difficult to check by legislation. Manufacturers are taking increased interest in the matter, and are learning that black smoke shows bad management. With mechanical stoking and careful watching, less smoke can be emitted from industrial chimneys and less coal burnt. But the truth is that the public generally, those who consume coal in factories and in their homes, are sadly ignorant of the waste caused by smoke. There is need for more steady propaganda on the subject, in order that any general legislation introduced may not be ahead of public opinion.

### RESCUE WORK.

SINCE the Armistice there has been a growing tendency in all departments of public health work to prevent overlapping and ensure co-operation. The formation of the Central Council for Rescue and Preventive Work in London a few weeks ago, as the result of a conference of representatives of public authorities, voluntary associations and local government officers, is another step in the right direction. Lord Astor has become the first president of the Council, Capt. C. E. Warburg, L.C.C., the first chairman, and Capt. L. F. Ellis and Mr. D. C. L. Ward, for the time being, joint honorary secretaries. All those connected with public health services in London realise that, owing to insufficient accommodation in reception homes or clearing-houses for women and girls needing special treatment, there have been widespread ravages of disease. This Council will be of the utmost importance and utility in providing information rapidly and bringing preventive and rescue work in London up to modern standards. Some other great cities might well follow London's example.

### HEALTH OFFICIALS.

A NEW Order, No. 276, issued by Sir Alfred Mond curtails the powers of local authorities with regard to the appointment of Medical Officers of Health and Sanitary Inspectors. For some years past the position of a keen M.O.H. has been weakened, owing to the fact that his position was to some extent dependent on the whims of his local council. Property owners on a Council have been known to object to slum improvements. There have also been cases of incompetent men being thrust into a municipal billet. It is hoped that this new order will remedy these weaknesses of our public health service. The qualifications of Medical Officers are now defined. Their appointments are subject to the Ministry's approval. Whole-time officials are to enjoy security of tenure, and can only be removed by the consent of the Minister of Health. This Order is very much to the good, and gives local officials credit for commonsense, specifying their duties under a few broad heads.

### THE SCHOOL MEDICAL SERVICES.

RECENTLY a ridiculous statement was made that it is proposed to transfer the school medical services to the Ministry of Health. Obviously such a transfer, even if desirable, is quite impracticable. How could the officials of a Government Department walk into a school controlled by a local authority which in its turn is responsible to quite a different Government Department, without endless friction resulting? At the present time there is close co-ordination between the Ministry of Health and the Board of Education, owing to the fact that Sir George Newman is Chief Medical Officer to both Departments. A working arrangement was established two years ago. In the majority of cases this is proceeding without any signs of friction. A somewhat unwise circular issued recently by an educational association caused a slight flutter, but of all the health services none is working more efficiently, more smoothly, or more satisfactorily.

### ANTHRAX FROM SHAVING-BRUSHES.

THERE have been too many cases of anthrax recently in Europe. Some have been traced to shaving brushes made in Japan, and others to hides. It is, however, satisfactory that an International Committee has now been appointed to inquire into the problem. How can wood and hair infected with anthrax spores best be disinfected? What are effective methods of preventing infection among flocks? Can infection from hides and skins be satisfactorily prevented? These are some of the questions that are to be discussed by this Committee, the chairman of which is Sir William Middlebrook, M.P., who was chairman of the Departmental Committee on the Prevention of Anthrax in 1913. The infection of wool was considered at the International Labour Conference at Washington in 1919 by the Committee on Unhealthy Processes, and again at Geneva last year.

### WOMEN STUDENTS AND THE LONDON HOSPITAL.

WE regret to notice the suggestion in a lay contemporary that the decision of the London Hospital to exclude women medical students should be made the subject of questions in Parliament. It is true that the London Hospital receives an additional grant from the University Grants Commission, and that this, with similar grants for special purposes, is contributed from public funds for medical education. But the grant is made unconditionally, and if women help to provide the money, the answer is that all taxpayers contribute to objects of which they do not approve, and were their approval necessary in each case, public funds could not be administered. At present some hospitals accept and some refuse women medical students. There are also schools reserved for them. This variety is all to their advantage; and why should the section of taxpayers which favours the admission of women students in every hospital in receipt of public grants for medical education be more privileged than that which takes another view? To follow the suggestion would injure the hospital. Even if compulsion were seriously in question, which it is not, the atmosphere created thereby would only recoil on the women for whose supposed benefit it is advanced.

### A WARNING TO TESTATORS.

THE difficulty which hospital secretaries have in persuading testators to define clearly the institutions that they wish to benefit was disastrously emphasised in the recent decision of the Chancery Division over a disputed legacy of £5,000 left by the late Mr. David Currie to the "Belfast Infirmary." The legacy was unsuccessfully claimed by the Royal Victoria Hospital, Belfast; and Colonel Gerald Hurst, M.P., who appeared on its behalf, vainly deposed that "outside London, and especially in the North, it was customary to call the principal hospital in the town supported by voluntary contributions 'the infirmary.'" The presumption that a testator was more likely to leave money to a charity than to an institution supported by the rates was set aside, and the Court declared that the words of the will clearly pointed to the Poor Law Infirmary of the Belfast Board of Guardians. The guardians were persons conducting a building which fulfilled all the purposes of a general hospital, and that building was called "The Belfast Infirmary." That accurately answered the description of the institution in the testator's will to which the legacy was given. Mr. Justice Russell's ruling will now create or confirm a precedent, and it becomes more important than ever that testators should clearly specify by the precise title the Institution which they intend to benefit. It is an interesting fact that, disputed cases apart, bequests are sometimes made to rate or tax-supported Institutions. But such bequests are rare, and in the interests of all alike, testators should be precise in their wording.

